# Stress-induced long-range ordering in spider silk

**DOI:** 10.1038/s41598-017-15384-8

**Published:** 2017-11-10

**Authors:** Johannes A. Wagner, Sandeep P. Patil, Imke Greving, Marc Lämmel, Konstantinos Gkagkas, Tilo Seydel, Martin Müller, Bernd Markert, Frauke Gräter

**Affiliations:** 1 Heidelberg Institute for Theoretical Studies (HITS), Heidelberg, 69118 Germany; 2 Heidelberg University, Institute for Theoretical Physics, Heidelberg, 69120 Germany; 3 RWTH Aachen University, Institute of General Mechanics, Aachen, 52062 Germany; 4 Helmholtz-Zentrum Geesthacht (HZG), Institute of Materials Research, Geesthacht, 21502 Germany; 5 University of Leipzig, Institute for Theoretical Physics, Leipzig, 04103 Germany; 6 Toyota Motor Europe NV/SA, Advanced Technology Division, Zaventem, 1930 Belgium; 7 Institut Max von Laue-Paul Langevin (ILL), Grenoble, 38042 France; 8 University of Kiel, Institute for Experimental and Applied Physics, Kiel, 24098 Germany; 9 Heidelberg University, Interdisciplinary Center for Scientific Computing (IWR), Heidelberg, 69120 Germany

## Abstract

The emergence of order from disorder is a topic of vital interest. We here propose that long-range order can arise from a randomly arranged two-phase material under mechanical load. Using Small-Angle Neutron Scattering (SANS) experiments and Molecular Dynamics based finite element (FE) models we show evidence for stress-induced ordering in spider dragline silk. Both methods show striking quantitative agreement of the position, shift and intensity increase of the long period upon stretching. We demonstrate that mesoscopic ordering does not originate from silk-specific processes such as strain-induced crystallization on the atomistic scale or the alignment of tilted crystallites. It instead is a general phenomenon arising from a non-affine deformation that enhances density fluctuations of the stiff and soft phases along the direction of stress. Our results suggest long-range ordering, analogously to the coalescence of defects in materials, as a wide-spread phenomenon to be exploited for tuning the mechanical properties of many hybrid stiff and soft materials.

## Introduction

A material’s mechanical behavior is ultimately defined by the underlying structure. Enormous progress has been made in the last decades in the knowledge and design of the molecular to macroscopic structure for even high-complexity materials, in order to tailor their mechanics and mechanical properties. However, our understanding of how the application of mechanical stress effects the material’s structure on the relevant length scales, even though at least similarly critical for mechanical performance, needs expanding.

Many composite materials of mechanical interest feature a coexistence of stiff and soft phases, in which the stiff phase reinforces and thereby strengthens the soft phase. Analogously, semi-crystalline polymers, including synthetic and biological materials, consist of a highly structured stiff phase alternating with an amorphous and comparably soft phase. It is well established that stretching semi-crystalline materials can lead to order on a short length scale, namely by stress-induced crystallization^1–4^. Amorphous chains straighten and align, and thereby self-assemble into more ordered structures under force. This also leads to a reduction in the average tilt angle of the backbones within crystallites relative to the fiber axis^1,2,5,6^. Thus, such materials feature different facets of stress-induced ordering on the scale of the individual chains and crystals. A completely unaddressed question, however, is if applied stress can also increase long-range order on the mescoscopic scale of a few nanometers in the distribution of stiff components within the amorphous matrix of composite or semi-crystalline materials, or of defects, respectively.

Interestingly, in a material with randomly distributed voids or defects, stress application causes these voids to nucleate. The coalescence and gradual volumetric growth of voids leads to the formation of larger defect-free regions and lowers the total internal energy of the system^7–9^. Similarly, stress can increase structural order in metals with irradiation-induced defects^10–12^. Each defect in a periodic arrangement of atoms or molecules gives rise to a local stress concentration. This stress can cause these defects to be ‘dragged’ towards each other, which lowers the total internal energy of the system. This results in larger defect-free regions separated by clusters of defects along the direction of stress application.

Protein crystals show a similar behavior: Zemlin *et al*. could show that upon irradiation beyond a critical threshold, defects vanish in finite sized protein crystals, resulting in stress-induced order due to the build-up of internal stresses^13^. They observed the same effect in regular 2D-lattices of macroscopic bubbles, corroborating their conclusion that mechanical stress and not thermal energy induced the order. These seminal experiments nearly two decades ago have not yet been attempted with other systems. Stress-induced order as a general principle has yet to be uncovered.

**Figure 1:**
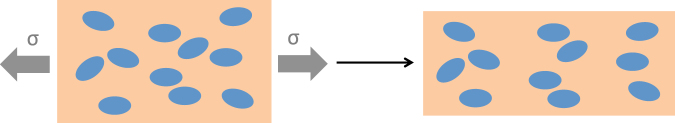
Scheme to illustrate the hypothesis of stress-induced order. Tensile loading can lead to ordering of the stiffer (blue) and softer (orange) components in a two-component system. The material builds up periodic density fluctuations featuring more soft regions with fewer stiffer particles (arrows), resulting in long-range order along the loading direction at a length scale larger than the stiffer components’ dimension.

Given that defects can be considered as a second phase in a two-phase system with inherently different mechanical properties, an obvious question is if stress-induced long-range order is a phenomenon similarly at play in composite or semi-crystalline materials (Fig. 1). An intriguing consequence of such ordering would be an attenuation of stress concentrations and an enhancement in toughness.

We here addressed this question for the nanoscale arrangement of crystallites within silk fibers. Silk is a protein-based high-performance semi-crystalline material. In contrast to synthetic semi-crystalline materials, crystalline regions are imprinted into the sequence, namely poly-alanine repeat units of a length of 6–10 monomers^14–16^. They alternate with glycine-rich disordered regions of roughly three times the length, which form the amorphous phase. The nanometer-sized crystalline units show a preferred orientation of the crystallites c-axis (chain direction) along the fiber direction^1,6,17,18^, but are otherwise thought to be randomly positioned^1^. Both, Small-angle neutron (SANS) and X-ray scattering (SAXS) experiments show a mesoscopic long period in fiber direction at a length scale similar to the length scale of individual crystallites, which currently lacks a structural interpretation^19–24^. Interestingly, we could previously show in a highly simplified finite element model that an ordered arrangement of crystallites, with regions of high and low crystallinity alternating along the fiber axis, would increase the fiber’s toughness^25^.

We here present results from a purely atomistically-informed (bottom-up) finite element model of a silk fiber under stress and small-angle neutron scattering (SANS) experiments of *Nephila edulis* dragline fibers. Both simulations and experiments unequivocally show an increase in long-range order of crystals along the fiber axis beyond the purely geometric effect of fiber elongation. Fluctuations of crystal density along the fiber axis increase upon stretching, thereby lowering mechanical energy. Our data suggests long-range order arising from mechanical work performed on the material to be vitally important to our understanding of silk fiber structure and mechanics, and potentially many other semi-crystalline or composite materials.

## Results and Discussion

### Crystals distribute periodically in strained silk fibers

**Figure 2:**
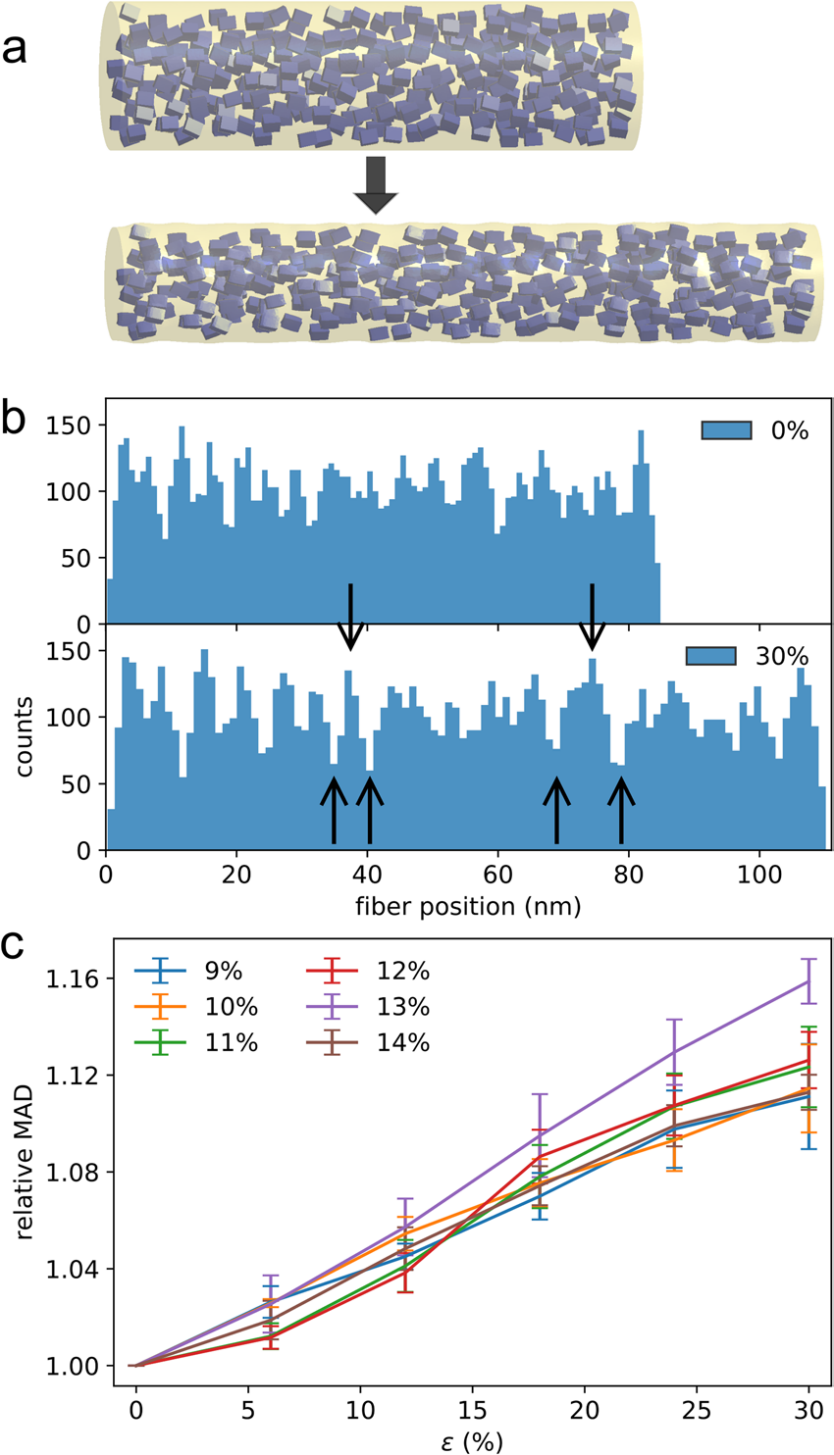
Increased long-range order in FE fiber models during loading. (**a**) A simplified 3D FE fiber model (see Suppl. Methods for details) with crystallites immersed into the amorphous phase was subjected to tensile load and the crystal distribution monitored. (**b**) Fluctuating crystallinity along the fiber axis of a fiber with 11% overall crystallinity for two different strains. Arrows exemplify cross-sections with increased amplitudes in crystallinity variations during loading. We note that even though this data representation does not straightforwardly reflect ordering, the quantification by MAD strongly suggests long-range ordering in all cases. (**c**) Mean absolute deviations (MAD) of crystallinity along the fiber axis as a function of external strain. For each overall crystallinity, values have been averaged over five individual fiber models. See Suppl. Figure 4 for the MAD of fibers with larger crystals and crystallinity.

To analyze potential stress-induced ordering in silk, we here resorted to a simplified two-phase finite element (FE) model of silk fiber comprising of the stiffer crystalline and the softer amorphous phase. We have developed this bottom-up FE model on the basis of atomistic MD simulations of *Araneus diadematus* silk protein^26,27^. We here present a novel refined FE model, which describes the amorphous phase as a viscoelastic and the crystalline phase as a plastoelastic material, of which the viscosity and yield stress again obtained from MD simulations^28–30^. Crystals were randomly distributed into the amorphous phase with the experimentally known FWHM for the tilt angle of 15° for native dragline silk of *A. trifasciata* ^18^ and *N. clavipes* ^1^ at crystallinities varying between 9 and 14% (Fig. 2a)^1^. We note that silk also features a phase of intermediate order, primarily composed of the glycine-alanine rich sequences flanking the poly-alanine repeats^1^, which extend the crystallites apparent dimensions and might also be the cause of the measured higher total crystallinity^1,18^. We therefore considered additional models with larger crystals and up to 17% crystallinity (Supplement Fig. 4). We applied constant strain rates to the fibers, and observed typical stress-strain curves with yield stress and strain, rupture stress and strain, and toughness values in agreement with experimental observations (see Supplementary Information, Suppl. Table 1), validating our simplified model.

Changes in structural order were also analyzed within the fiber under load. Figure 2 shows the crystallinity along the fiber axis for different strain values. The initial random packing transforms into a crystal distribution with pronounced peaks of higher crystallinity upon stretching (Fig. 2a). The increase in crystallinity fluctuations was quantified by summing up the deviations from the mean crystallinity along the fiber axis (Fig. 2b,c). We always observed a significant increase in these fluctuations upon straining. These fluctuations roughly lie in the 4–8 nm range, i.e. beyond the length of single crystals along the fiber axis, which here was chosen to fall in line with the poly-alanine repeat lengths of 2.7 nm^27^. This advocates a stress-induced ordering. The significantly larger increase in MAD for a crystalinity of 13% might originate from the packing routine for this specific volume and size. ratio of crystal and fibers, or is simply a consequence of insufficient sampling. We observe the same increase in MAD upon straining the fiber for models with larger crystal sizes and overall crystallinity (Suppl. Fig. 4) or with zero tilt angle (Suppl. Fig. 5), suggesting our finding to be robust with regard to these structural parameters.

### SANS confirms stress-induced order in silk

**Figure 3:**
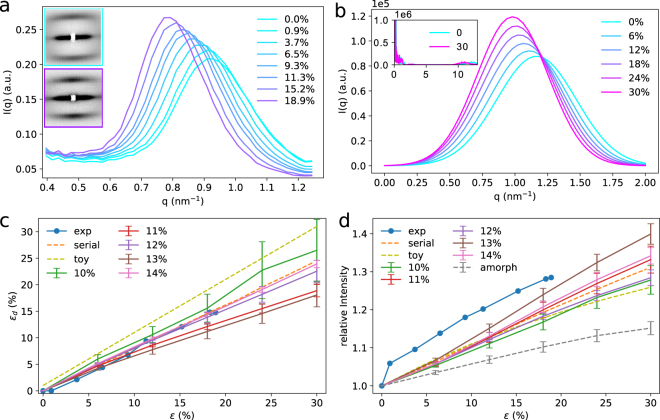
Scattering intensities from SANS and FE corroborate stress-induced long-range order. (**a**) Meridional long period peaks of *I*(*q*) recorded by SANS on a D_2_ O humid spider silk sample for varying strain values *ε* in % elongation. Inset: 2D scattering image at no strain (top) and maximum strain (bottom), respectively. The peak position shifts towards lower *q* with increasing tensile load. In addition, the peak becomes slightly sharper. The yellow arcs represent the azimuthal integration angle of 45°. (**b**) Long period peaks of *I*(*q*) calculated from FE calculations of a strained fiber model of 11% crystallinity. Inset: *I*(*q*) over a larger *q*-range also including peaks from intra-crystallite scattering. (**c**) Small-angle strain *ε*_*d*_ as manifested by the relative change in the long period peak position versus macroscopic fiber strain *ε*, calculated from SANS (**a**) and the FE model (**b**). (**d**) Relative increase of scattering intensity, *I*(*q*)/*I*_*ε*=0_(*q*), as recorded by SANS or calculated for FE models of different crystallinity. As a reference, the intensity increase predicted by a simplified analytical model (see Suppl. Methods), by a fully amorphous model, in which the originally crystalline parts still yield the scattering contrast, and by a perfectly ordered serial model is also shown. (Note: the relative intensity increase from the analytical model is a function of the number of scatterers N, but this dependency vanishes for large *N*. We chose *N* = 60 in Eq. 1, which is large enough to avoid this dependency.

Small-angle neutron scattering (SANS) was undertaken to test our computational prediction of stress-induced ordering in silk fibers. Deuterated fiber bundles using H/D exchange of *Nephila edulis* spider silk^23,31^ were subjected to increasing values of constant strains, for each of which a SANS pattern was recorded. An increase in scattering peak intensity on the nanometer length scale upon fiber stretch would reflect a long-range order of crystalline units within the flexible and thus more strongly deuterated amorphous phase, which also includes a partially ordered phase around the non-deuterated crystallites^32^. The two-dimensional SANS patterns (inset in Fig. 3a) show the distinct meridional peaks as an indication of the mesoscopic long period in fiber direction previously reported^19–24,33^ as well as a strong equatorial streak. The latter would yield additional information on the lateral (perpendicular to the fiber axis) arrangement of the nanocrystals in the spider silk fiber; however, in the context of the ordering effect in fiber direction, the respective analysis is not relevant and will be discussed elsewhere. Upon tensile load, the 2D SANS pattern exhibits two obvious changes: the position of the meridional peaks moves towards smaller scattering angles and the peaks become more pronounced. The integration of the two meridional peaks (symmetrically above and below the equator) over a 45° azimuthal angle each result in radial intensity distributions *I*(*q*) where *q* is the modulus of the wave vector transfer defined as $q=\frac{4\pi }{\lambda }\,\sin \,\theta$ with the scattering angle 2*θ*. *I*(*q*) measures correlations of distances $d=\frac{2\pi }{q}$ along the fiber axis between less deuterated regions, which here are the highly ordered *β*-sheet rich crystal units. The respective *I*(*q*) curves are shown in Fig. 3a for different values of the macroscopic strain *ε*. The second order peak (at 2*q*) is extremely weak, which is consistent with SAXS experiments on wet silk fibers^20^. As already seen in the 2D pattern, the long-period peak intensity increases and shifts to smaller *q* upon stretch.

The increase in *I*(*q*) of the long period observed in the SANS experiment is in line with the observations from the FE silk fiber model (Fig. 2). For a more direct comparison, we calculated the SANS observable, *I*(*q*), from the fiber model. More precisely, *I*(*q*) was obtained from the ensemble averaged correlations of distances $d=\frac{2\pi }{q}$ between the mesh nodes within and between the crystals (Fig. 3b, see Suppl. Information for details). As expected, the finite size of the model causes high intensities below 1 nm^−1^, and correlations within the crystalline units result in high intensities in the *q* = 10–13 nm^−1^ range (Fig. 3b, inset). We can also recover the long period, i.e. the correlations of inter-crystal distances along the fiber axis, as observed in the SANS experiments, with high *I*(*q*) in the range of 1 nm^−1^. We note that quantitative comparisons of absolute *I*(*q*) values between SANS experiments and FE simulations are not possible, because the number of scatterers in the experiment are unknown. As in the SANS experiments, the maximum of *I*(*q*) increases with strain and moves towards smaller *q* upon stretching.

For both, the SANS and FE long period, the *q*-position of the peak maximum was then used to calculate the corresponding *d*-spacing. Taking the value at no strain as reference ${d}_{0}=d(\varepsilon =\mathrm{0)}$, we can define the microscopic small-angle strain as ${\varepsilon }_{d}(\varepsilon )=\frac{d(\varepsilon )-{d}_{0}}{{d}_{0}}$. The strain dependence of *ε*_*d*_ is plotted in Fig. 3c and is clearly linear with a ratio of 0.86 (SANS) or 0.85–0.91 (FE) between *ε*_*d*_ and *ε* at maximal experimental *ε*. A value < 1 is associated with deformation by fibril slip^34^, which is consistent with our simulation results assuming lateral friction within the amorphous phase of spider silk^29^. The lateral friction within the amorphous phase is connected to the increased unraveling of the random polymer chains upon strain, which is be a process associated with friction and thus reflected in the spectroscopic response^29,31,35^.

Figure 3d shows the stress-induced change in the intensity maximum. In both the SANS experiments and FE calculations, independent from the initial arrangement and crystallinity of the model, we obtained a significant stress-induced increase in *I*(*q*) of the long period. This finding indicates increased order of the periodic arrangement of the long period structure. To distinguish such stress-induced ordering from a purely geometric effect, we calculated the strain-dependent long periods also for a fictitious reference system, namely a homogeneous fiber with identical viscoelastic properties throughout (Fig. 3d). In this case, the intensity of the long-period also steadily increases upon loading, but to a significantly smaller extent than in the SANS experiment or in the actual FE silk fiber models. Remarkably, the extent of ordering we observe in our silk fiber models is as high as the stress-induced ordering expected for a toy model of an already completely pre-ordered fiber. In this simple model, patches of high crystallinity move away from one another under tensile stress in an affine deformation. The toy model qualitatively confirms the strain-dependence of the position and height of the long-range peak observed in FE calculations and SANS experiments (Fig. 3c,d and Supplementary Methods):

1 $${q}_{{\rm{\max }}}\sim 2\pi /\mathrm{(1}+\varepsilon ),\quad S({q}_{{\rm{\max }}})\sim 1+N\mathrm{[1}-{\mathrm{(2}\pi \sigma )}^{2}\mathrm{/(1}+\varepsilon {)}^{2}]\quad \quad \mathrm{(0 < }\varepsilon \ll \mathrm{1)}$$

Taken together, the combined results from experiments and simulations strongly support the notion of stress-induced ordering of the crystalline phase along spider silk fibers, which relies on the fact that one component is significantly stiffer than the other, resulting in a phase separation. Regions rich in amorphous phase can more readily expand separating crystal-reinforced regions from one another and minimizing mechanical energy. While experiments have been performed with supercontracted silk, our more general computational and analytical models suggests that ordering can be at play in both supercontracted and native silks. The independence of our results with respect to crystal tilting, which is more pronounced for supercontracted silk, corroborates this notion. The striking quantitative agreement between the SANS experiments and the FE simulations, which have been performed independently and on different spider silks, hint towards that long-range ordering is a general phenomenon not depending on intricate details of specific silks such as exact tilting of crystals, sequence of the amorphous phase, chain connectivity, or crystal size and distribution. Yet, we only have started to assess how much these factors influence the extent of ordering, by varying some of the model parameters (tilt angles, crystal sizes, crystallinity). The simplicity and versatility of our model, however, opens new roads towards deciphering the determinants of mesoscopic order in silk, being supercontracted or dry, dragline or not, from spiders or bombyx mori.

## Conclusions

By combining simulations and experiments, we here show that tensile forces acting on systems with two mechanically distinct phases can lead to an increase in long-range order. The ordering goes significantly beyond a purely geometric effect, which solely arises from the lengthening and thinning of the specimen. Remarkably, we obtained a striking quantitative agreement between the finite element simulations, the SANS experiments and a simple analytical model. Together, this strongly advocates stretch-induced ordering in silk, and possibly as a general principle of any two-phase system, being it semi-crystalline or a composite, and at varying length scales of the two phases, from the nanometer to micrometer scale or even beyond. Whenever one of the two phases is much softer than the other (or actually a defect^13^), the softer phase will more readily extend in regions less stiffened by the other phase, resulting in a mechanically induced phase segregation. We also show that stress-induced long-range ordering is largely independent from the extent of crystallinity fluctuations at zero stress. It does rely, however, on some pre-existing fluctuations in the distribution of crystals, such that order should not arise from a fiber with a perfectly homogenous crystal distribution. The observed ordering effect is in line with the very recent observation by quasi-elastic neutron scattering experiments of spider silk that the diffusive mobility of the amorphous phase increases with tensile strain^31^. We propose that tensile forces present during the spinning process of silk or other synthetic fibers enhance long-range pre-order, which in turn boosts further ordering during tensile loading. The parameter range of volume ratios, pre-order, and mechanical properties of the two phases within which stress-induced ordering is at play remains to be systematically quantified. Given that the toughness of a fiber is maximal for a highly ordered arrangement^26^, lowering the friction between the soft and stiff components of a hybrid material might improve ordering and thereby toughness, another important point of future studies. We envision a wide range of materials, from nano-composites to block-copolymers, for which stress-induced long-range order can be exploited as a mean to tune mechanical properties.

## Materials and Methods

The reported SANS experiments were performed for a fiber bundle of *Nephila edulis* spider dragline silk at different strains. The fibers were humidified with D_2_ O, and the scattering contrast arose from the H/D-contrast between the D_2_ O-accessible disordered (amorphous) regions and the crystallites. The latter are much less accessible for D_2_ O and therefore do not undergo an H/D-exchange. A novel bottom-up finite element (FE) fiber model for *Araneus diadematus* spider dragline silk based on previous atomistic Molecular Dynamics simulations^25,26,29^ was subjected to constant strain rates using LS-DYNA. All computational and experimental details are given in the Supplementary Methods.

## Electronic supplementary material

Supplemental Information
