# Supplementary material for: Stress-induced long-range ordering in spider silk
Source: Sci Rep. 2017 Nov 10;7:15273. doi: 10.1038/s41598-017-15384-8 (PMC5681667; doi:10.1038/s41598-017-15384-8)
Supplement: Supplementary file 1 — Supplemental Information [file 41598_2017_15384_MOESM1_ESM.pdf]

Supplementary Material Stress-induced long-range ordering in spider silk

Johannes A. Wagner[1,2], Sandeep P. Patil[3], Imke Greving[4], Marc Lämmel[5], Konstantinos Gkagkas[6], Tilo Seydel[7], Martin Müller[8], Bernd Markert[3], and Frauke Gräter[1,9,*]

[1]Heidelberg Institute for Theoretical Studies (HITS), Heidelberg, 69118, Germany
[2]Heidelberg University, Institute for Theoretical Physics, Heidelberg, 69120, Germany
[3]RWTH, Institute for General Mechanics, Aachen, 52062, Germany
[4]Helmholtz-Zentrum Geesthacht (HZG), Institute of Materials Research, Geesthacht, 21502, Germany
[5]University of Leipzig, Institute for Theoretical Physics, Leipzig, 04103, Germany
[6]Toyota Motor Europe NV/SA, Technical Center, Zaventem, 1930, Belgium
[7]Institute Max von Laue-Paul Langevin, Grenoble, 38042, France
[8]University of Kiel, Institute for Experimental and Applied Physics, Kiel, 24098, Germany
[9]Heidelberg University, Interdisciplinary Center for Scientific Computing (IWR), Heidelberg, 69120, Germany
*frauke.graeter@h-its.org

ABSTRACT

Finite element modelling

Model building

Geometry

The geometry of the fiber defines the positions and arrangement of the crystals inside the amorphous matrix of the cylinder shaped fiber. We created fiber models with a length of 85 nm and a diameter of 22 nm, which roughly corresponds to half a nano-fibril in length[1] and diameter[2]. We varied the crystallinity from 9 – 14 volume %, which is consistent with experimental observations[3,4].

The crystals were modeled with a size of $1.9 \times 2.0 \times 2.7\,\mathrm{nm}^3$ in interchain (H-bonds), intersheet and chain direction (fiber axis) as defined in previous FE studies of silk based on the poly-alanine repeat unit sequence length[5–7]. We additionally created a fiber model with larger crystals of size $4.2 \times 2.3 \times 5.2\,\mathrm{nm}^3$ based on WAXS measurements of Warwicker classification $\beta(3/5)$ spider silks[8]. In the later calculation of SANS data from our model (see below), we will treat the crystals as scatterers, thus assuming H/D exchange to be limited to the regions around these crystals. One can therefore consider the model with smaller crystals to reflect the situation in which the highly ordered inner cores of the crystallites do not exchange but the adjacent partially ordered regions made from glycines/alanine rich sequences do undergo H/D exchange[9]. The larger crystal sizes of the second model, instead, reflect the other extreme, in which this partially ordered phase exchanges only marginally, i.e. is involved in scattering as well.

The crystals were then randomly placed inside the fiber by a packing routine that only obeyed a minimum inter-crystal and crystal-to-fiber surface distance. Lastly, the crystals were aligned along the fiber axis and tilted with a normal distributed angle with a FWHM of 15°, as observed experimentally[3,4]. Since we only allow randomly placed, rotated and tilted crystals to be included that fullfil the minimum distance criteria, the resulting tilt angle distribution is not normal distributed anymore. Therefore we only report the average tilt angle for the fibers.

In addition, as a reference, a model with a serial arrangement of crystal slices and an overall crystallinity of 24.7 % was created with alternating cross-sections, which were either fully crystalline or fully amorphous[5].

Meshing and convergence

The fiber geometry was meshed using GMSH 2.13.2 and the in-built unstructured 3D-Delaunay tetrahedral mesh algorithm[10], which is well suited for creating a coherent interface mesh between the amorphous and randomly embedded crystalline parts. The mesh was then optimized by deploying the NETGEN algorithm[11], which increases element quality by displacing, reconnecting and/or removing nodes (Suppl. Fig. 1). The final mesh quality was evaluated by monitoring distributions of three criteria for tetrahedral element quality:

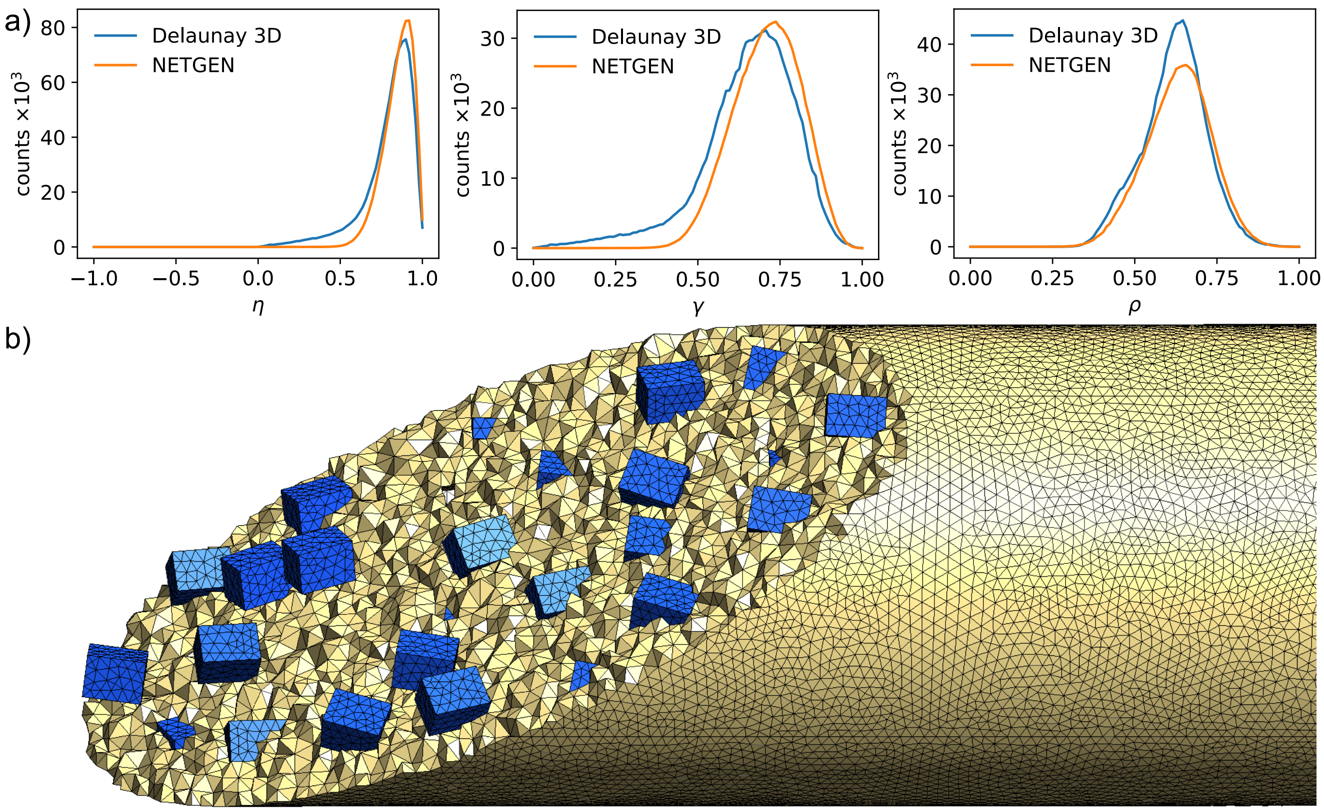

Figure 1. Mesh quality. (a) Distributions before (blue) & after (orange) NETGEN optimization for a representative fiber (10% crystallinity). The elements with the worst quality, *i.e.* lowest η, γ and ρ values were significantly reduced after optimization. While the average values increased. Before optimization: $\gamma = 0.7847$ & $\rho = 0.6504$, after optimization: $\gamma = 0.8437$ & $\rho = 0.7085$. (b) Cross section of meshed fiber with embedded crystals.

- η, the volume of the element to the power of 2/3 divided by the squared sum of the edge lengths.
- γ, the volume divided by the summed face areas and the maximal edge length.
- ρ, the minimum edge length divided by the maximum edge length of the tetrahedron.

The tetrahedral elements were defined as LS-DYNA element formulation 13, a 1 point constant stress element with nodal pressure averaging, which significantly alleviates volumetric locking compared to element formulation 4. To obtain the maximum possible mesh resolution that still yields accurate results, the characteristic length of a finite element (l_c) was varied over the range of 0.45 to 1.2 nm (Suppl. Fig. 2). Fiber models with different mesh resolutions in this l_c range were pulled with a strain rate of $0.0175\,s^{-1}$ to a maximum strain of 1.75 % (strain velocity of 1.4875 nm/ns for a 85 nm long fiber) and the terminal stress was obtained. This terminal stress should converge with decreasing l_c. The highest l_c value for the converged stress was then used for the production simulations.

Parametrization

For the amorphous phase, a viscoelastic material model was used. Parameters for the density, elastic modulus, and viscosity were obtained by a bottom-up approach using Molecular Dynamics simulations of a representative atomistic building block of the amorphous phase as described previously[12]. The crystalline phase was modelled as a plastoelastic material, with a density, elastic modulus, and yield stress again obtained from Molecular Dynamics simulations as published earlier[5–7, 13]. All underlying Molecular Dynamics simulations were performed for representative sequences from *Araneus diadematus* spider silk. We emphasize that this geometry and parametrization results in a highly simplified model of spider silk, as it considers only two phases: a fully crystalline and fully amorphous phase. Importantly, by design this model neglects partially crystalline intermediate regions[3, 14–17] and other structural units such as the debated polyproline-II helices[18–22]. It also ignores any chain connectivity between the amorphous

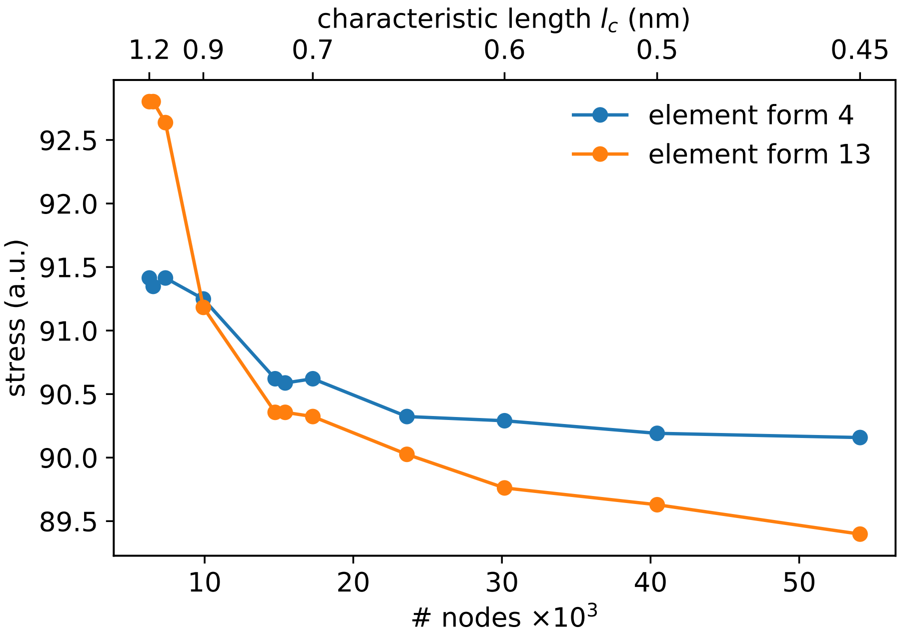

Figure 2. Mesh convergence for tetrahedral element formulations 4 & 13. The terminal stress after straining (1.75 %) is plotted against the number of nodes, which is determined by the characteristic length of the meshing algorithm. A value of $l_c = 0.55$ nm and element formulation 13 were used for production runs.

and crystalline phases, as we here aim to address a general phenomenon that is likely present in any two-phase material with stiff and soft components.

As another reference model that does not feature differences in the mechanical properties of the two phases, an all-amorphous model was used with a geometry and mesh identical to the fiber described above but with parameters of the amorphous phase throughout the whole fiber. In this case, the geometry is only relevant for the definition of scatterers (see below) but irrelevant for the tensile test.

Computational tensile tests

All finite element calculations were carried out using LS-DYNA R8.1.0 and results visualized and processed with LS-Prepost 4.3 as well tools developed in Julia and Python[23,24]. The fibers were subjected to a constant strain rate of $0.1\,\mathrm{s}^{-1}$, which was comparable to the range used in experiments[25,26], up to a maximum strain of $\epsilon = 0.5$. The corresponding SANS experiments were done in quasi-equilibrium, but slow strain rates have been shown to expose any significant differences in the stress-strain behaviour[26,27]. Nodal positions and von-Mises nodal stresses were written out at a rate of $0.02\,\mathrm{s}^{-1}$. As boundary conditions, the nodes of one cylinder surface were fixed, and the surface nodes on the other side were subjected to load. Fiber rupture was defined at the point when more than 5% of all crystal nodes exceeded the yield stress and underwent plastic deformation (Suppl. Figs. 6,7,8). We note that our model does not predict the strain-induced stiffening often observed in experiments[28], as the model does not capture the finite chain length of proteins in the amorphous phase nor the increase in crystallinity under tensile load (Suppl. Fig. 6).

Calculation of $I(\mathbf{q})$ from FEM model

The scatter intensity is defined as $I(\mathbf{q}) = P(\mathbf{q}) \cdot S(\mathbf{q})$ with $P(\mathbf{q})$ being the form factor intensity and $S(\mathbf{q})$ the structure factor. These in turn are defined as

$$P(\mathbf{q}) = \left| \sum_i^N \mathrm{e}^{i\mathbf{q}\cdot\mathbf{r}_i} \right|^2 \tag{1}$$

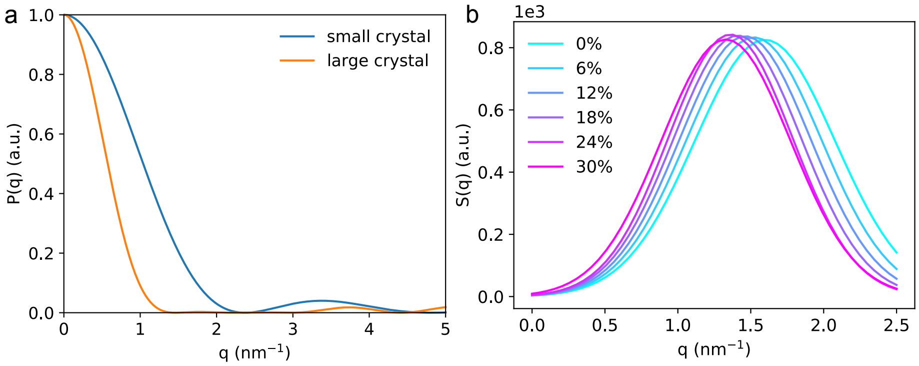

Figure 3. (a) Form factor intensity $P(|\mathbf{q}|)$ in direction of the fiber axis at zero strain for the two different crystal sizes, which remains mostly unchanged for increasing external strain. (b) Examplary structure factor $S(|\mathbf{q}|)$ of a fiber with 14% crystallinity for different strains, obtained from the geometrical centers of the crystallites. Due to significantly less data points for the structure factor, the gaussian fitting routine is only very limited usable. Both, form and structure factor were calculated from the FE model.

and

$$S(\mathbf{q}) = \left\langle \sum_{j,k}^{N} \mathrm{e}^{i\mathbf{q}\cdot(\mathbf{R}_j - \mathbf{R}_k)} \right\rangle \tag{2}$$

where $\mathbf{r}_i$ are scatterer positions within a single crystal, and $\mathbf{R}_{j/k}$ are the crystal positions. This is similar to the WAXS pattern calculations previously done by Ulrich et. al.[29]. Rotational averaging and angular integration were taken into account by averaging $S(\mathbf{q})$ over the rotation $\mathbf{A}(\zeta)$ of the angle around the fiber axis ζ and angular integration over the rotation $\mathbf{B}(\chi)$ of the azimuthal angle χ:

$$S(\mathbf{q}) = \frac{1}{K \cdot L} \sum_{\zeta_k \in [0,2\pi)}^{K} \sum_{\chi_l \in [0,\pi/4]}^{L} \sum_{i,j=0}^{N} \mathrm{e}^{i(\mathbf{A}^{\mathrm{T}}\mathbf{B}^{\mathrm{T}}\mathbf{q})\cdot(\mathbf{R}_i - \mathbf{R}_j)} \tag{3}$$

with K $= 2\pi/\Delta\zeta$ and L $= \pi/4\Delta\chi$ as number of angular averaging and integration steps. The experimental small-angle neutron scattering (SANS) data were angular integrated with an azimuthal angle of $\chi = \pi/2$, likewise for the calculated data.

Analogous to the experimental scatterers, which are the non-deuterated hydrogen atoms inside the crystals (or in the inner core of the crystals[30], we defined the mesh's crystal nodes as scatterer positions. Thus, for our FE models, the crystals' form factor can be easily calculated and isolated from the structure factor and its effect on the overall intensity can be quantified.

The form factor for our crystals of size $1.9 \times 2.0 \times 2.7\,\mathrm{nm}^3$ and $4.2 \times 2.3 \times 5.2\,\mathrm{nm}^3$ in the range of q-values of our interest is shown in Suppl. Fig. 3a. The structure factor itself shows only marginal changes upon straining (Suppl. Fig. 3b), and it is the product of the form and structure factor that leads to the observation of an intensity increase in the FE model, and thus likely also in the SANS experiments. In other words, it is the stiffness of the crystals and the high extensibility of the amorphous phase that give rise to the intensity increase, and this effect is lost when considering only crystal centers (*i.e.* structure factor), or, in the analytical toy model, delta functions instead of Gaussians. In our analysis, we directly compared the experimental and calculated intensities to one another.

Analytical toy model

General transformation of $S(\mathbf{q})$ under deformation. Under a general deformation $\mathbf{f}$, which gives the new position $\mathbf{r}' = \mathbf{f}(\mathbf{r})$ of a particle that was initially located at $\mathbf{r}$, the structure factor $S(\mathbf{q})$ transforms as

$$S'(\mathbf{q}) = 1 + n' \int \mathrm{d}\mathbf{r}\, h'(\mathbf{r}) \mathrm{e}^{i\mathbf{q}\cdot\mathbf{r}} = 1 + n' \int \mathrm{d}\mathbf{r}\, \det[D\mathbf{f}] h(\mathbf{r}) \mathrm{e}^{i\mathbf{q}\cdot\mathbf{f}(\mathbf{r})}, \tag{4}$$

because the total correlation function after the deformation is given by $h'(\mathbf{r}) = h[\mathbf{f}^{-1}(\mathbf{r})]$. For an affine deformation $\mathbf{f}(\mathbf{r}) = \Lambda \cdot \mathbf{r}$, where the matrix Λ itself does not depend on the position $\mathbf{r}$, the average density n' of the deformed solution is given by $n' = n/\det\Lambda$ and Eq. (4) therefore becomes

$$S'(\mathbf{q}) = 1 + n\int \mathrm{d}\mathbf{r}\, h(\mathbf{r})\mathrm{e}^{i(\Lambda^{\mathrm{T}}\cdot\mathbf{q})\cdot\mathbf{r}} = S(\Lambda^{\mathrm{T}}\cdot\mathbf{q})\,. \tag{5}$$

This implies that the peak positions of the structure factor are changed, whereas the peak heights remain unchanged upon deformation. An extension of magnitude ϵ, *i.e.*, $\Lambda(\epsilon) = \mathrm{diag}[1+\epsilon, 1-\nu\epsilon, 1-\nu\epsilon]$ with Poisson ratio ν, for instance, characterized by its magnitude ϵ in x-direction and the Poisson ratio ν, one obtains that the strain-dependent structure factor reads $S(q,\epsilon) = S[(1+\epsilon)q, 0]$ if the scattering vector is parallel to the stretch direction, *i.e.*, $\mathbf{q} = q\mathbf{e}_x$.

Scattering from a fiber. To investigate the scattering from a stretched silk fiber, we assume that the fiber is parallel to the x-axis and that the incoming beam points into the y-direction. Exploiting the systems symmetry, we use spherical and cylindrical coordinates

$$\mathbf{q} = q\begin{pmatrix}\sin\gamma\\ \cos\gamma\\ 0\end{pmatrix} \quad \text{and} \quad \mathbf{r} = \begin{pmatrix}x\\ \rho\cos\varphi\\ \rho\sin\varphi\end{pmatrix}, \tag{6}$$

for the scattering vector and the particle-particle distance vector, respectively, where is is assumed that the scattering profile is recorded in the x-y-plane. In this way the azemuthal angle of $\mathbf{q}$ can be set to 0. The polar angle γ between $\mathbf{q}$ and the incoming beam is related to $q \equiv |\mathbf{q}|$ and the wave number k of the beam as

$$q = 2k\cos\gamma\,, \tag{7}$$

which can be derived as follows. The wave vectors $\mathbf{k}_{\mathrm{i}}$ and $\mathbf{k}_{\mathrm{s}}$ of the incoming and the scattered wave have both the same absolute value k and differ in their direction by the scattering angle θ, *i.e.*, $\mathbf{k}_{\mathrm{i}}\cdot\mathbf{k}_{\mathrm{s}} \equiv k^2\cos\theta$. The length q of the scattering vector $\mathbf{q} = \mathbf{k}_{\mathrm{s}} - \mathbf{k}_{\mathrm{i}}$ is thus related to θ as $q^2 = 2k^2(1-\cos\theta)$. Inserting these relations into the defining equation $\cos\gamma \equiv -\mathbf{q}\cdot\mathbf{k}_{\mathrm{i}}/(qk) = (k/q)(1-\cos\theta)$ of the polar angle γ, we obtain Eq. (7).

From the parametrization in Eq. (6) follows $\mathbf{q}\cdot\mathbf{r} = q(x\sin\gamma + \rho\cos\gamma\cos\varphi)$ and with $h(\mathbf{r}) = h(x,\rho)$, which is independent of φ due to the rotational symmetry of the system around the fiber axis, we obtain

$$\begin{aligned} S'(\mathbf{q}) \equiv S(\mathbf{q},\epsilon) &= 1 + n\int \mathrm{d}\mathbf{r}\, h(x,\rho,\epsilon)\mathrm{e}^{i\mathbf{q}\cdot\mathbf{r}} \\ &= 1 + 2\pi n\int \mathrm{d}x \int \mathrm{d}\rho\,\rho h(x,\rho,\epsilon) J_0\left[q\rho^2/(2k)\right]\cos\left[qx\sqrt{1-q^2/(4k^2)}\right] \\ &\sim 1 + 2\pi n\int \mathrm{d}x \int \mathrm{d}\rho\,\rho h(x,\rho,\epsilon)\cos(qx)\,, \end{aligned} \tag{8}$$

where the asymptotic scaling in the third line applies for $q \ll k$. In the second line, we performed the integral over φ, used Eq. (7) to substitute q for γ, and removed the imaginary contributions exploiting that the result is invariant under the transition $x \mapsto -x$. For the silk experiments, the neutron beam has a wavelength of $2\pi/k = 0.6\,\mathrm{nm}$, the considered peak is located around $q \approx 0.1\,\mathrm{nm}^{-1}$, yielding a sufficiently small ratio $q/k \approx 0.01$ (corresponding to $\theta \approx 0.8°$), thereby justifying the use of the asymptotic relation in Eq.(8).

Stretch-dependent scattering from a pre-ordered fiber. To investigate how stretching affects the scattering from the fiber, an integrated total correlation function is introduced:

$$h(x,\epsilon) \equiv (2\pi nL/N)\int \mathrm{d}\rho\,\rho h(x,\rho,\epsilon)\,, \tag{9}$$

so that the structure factor in Eq. (8) takes the more simple form

$$S(q,\epsilon) = 1 + (N/L)\int \mathrm{d}x\, h(x,\epsilon)\cos(qx)\,, \tag{10}$$

which suggests the toy model approach outlined below.

We describe the silk fiber as a one-dimensional chain of scatterers, obtained by projecting the three-dimensional scatterer positions onto the center line of the fiber. Their coordinates x along the fiber axis represent the interfaces between the matrix and the embedded crystallites. The function $h(x,\epsilon)/L$ in Eq. (10) then the distribution of the scatterer distances x. The structure of the silk material is characterized by spatial domains of high crystallite density.

The mean distance between neighboring domains is set to 1 and denotes their size (*i.e.*, the length along the fiber axis) by 2σ. Coherent scattering from objects placed in two adjacent domains thus generates a peak of the structure factor around $q = 2\pi$. In the following, we address the influence of fiber stretching on the position and shape of this (first-order) domain-domain peak. The more densely packed conglomerates within the crystallite-rich domains are stiffer, while the matrix between the domains is more easily stretchable. In a first approximation, one can therefore take the relative positions of the scatterers within the domains to be conserved and the inter-domain distances to follow an externally imposed strain affinely.

Within our toy model approach, we represent the two neighboring domains by two Gaussian peaks in the scatterer density, which yields the following distribution of the scatterer distances:

$$h(x,\epsilon)/L = \frac{1}{4\sigma\sqrt{2\pi}}\left[2\mathrm{e}^{-\frac{x^2}{2\sigma^2}} + \mathrm{e}^{-\frac{(x+1+\epsilon)^2}{2\sigma^2}} + e^{-\frac{(x-1-\epsilon)^2}{2\sigma^2}}\right]. \tag{11}$$

The ϵ-dependent peak positions account for the affine elongation of large distances between scatterers located in different domains, while the conservation of the scattering geometry within the same domain is represented by the ϵ-independent peak width σ. Inserting this distribution into Eq. (10), the peak of the structure factor for the stretched chain is as follows:

$$S(q,\epsilon) = 1 + N\cos^2\left[\frac{(1+\epsilon)q}{2}\right]\mathrm{e}^{\frac{-\sigma^2 q^2}{2}}. \tag{12}$$

The toy model qualitatively confirms the strain-dependence of the position and height of the long-range peak observed in FE calculations and SANS experiments (Fig. 3 of main text):

$$q_{\max} \sim 2\pi/(1+\epsilon), \quad S(q_{\max}) \sim 1 + N[1-(2\pi\sigma)^2/(1+\epsilon)^2] \qquad (0 < \epsilon \ll 1) \tag{13}$$

Small-Angle Neutron scattering experiments

The *Nephila edulis* spider silk sample with a total mass at ambient humidity of $m_n = 104\,\mathrm{mg}$ was obtained by the forced silking technique[31] with a reeling speed of 10 mm/s. The sample consisted of a few uninterrupted dragline fibers wound around a pair of steel hooks with an initial gap between the inner edges of approximately 4 cm. The sample thus formed a bundle with a width of approximately 1 cm and a thickness of 2.0×2.7 mm. It had previously been used in a neutron spectroscopy experiment (immediately prior to the experiment), and the fibers were therefore previously strained and relieved from strain before the experiment.

The SANS experiments were carried out using the beam line D11 at the high-flux research reactor of the ILL in Grenoble, France, using a wavelength of $\lambda = 6\,\text{Å}$. The beam size was defined by a rectangular slit 7 mm wide and 10 mm high, and the reported SANS patterns were recorded at a detector distance of 4 m.

The silk sample was mounted on a computer-controlled tensile machine[17,32] inside a hermetically sealed chamber at ambient temperature with the silk fiber bundle axis vertically aligned. Within the sample chamber, a humid atmosphere saturated with heavy water (D_2O) vapor was set by filling small troughs located at the bottom of the chamber with heavy water. The saturated (100 % RH) humidity level and temperature inside the chamber were monitored by adequate sensors.

SANS patterns were recorded at constant elongations, and the elongations between recordings were carried out at a constant strain rate of $0.000\,21\,\mathrm{s}^{-1}$ (0.01 mm/s, see supplement of Krasnov *et al.*[17]). Patterns were recorded immediately after reaching each new elongation with an integration time of 1 min. As soon as a stable SANS pattern was reached, the patterns were integrated over a 40 min recording time. We note that absolute intensities of experiments and simulations can not be straightforwardly compared, given that the dead time and sensitivity of the detector as well as contrast variation influence $I(\mathbf{q})$.

A difference between the observations from SANS and FE calculations (see above) due to the two different spider species used for experiments (*Nephila edulis*) and for computational modelling (*Araneus diadematus*) can not be excluded. However, the modelled dragline silk shares the same size of poly-alanine repeats and has a very similar sequence composition to the silk used in experiments[28,33,34]. We believe this to be of minor importance due to the

high abstraction of translating a (slightly varying) sequence of the amorphous phase into a single viscosity parameter in the FE model. Our simplified and rather generally valid FE model is therefore unlikely to capture the overall minor sequence differences.

Scattering intensities of the long period signal of the crystallites were obtained as a function of strain and compared to those obtained from SANS.

Supplementary Figures

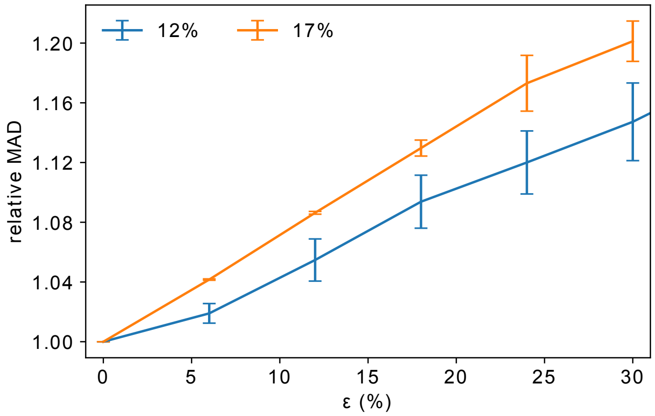

Figure 4. MAD evolution upon straining for fibers with large crystals ($4.2 \times 2.3 \times 5.2\,\mathrm{nm}^3$) with a crystallinity of 12 and 17%, 5 simulations each.

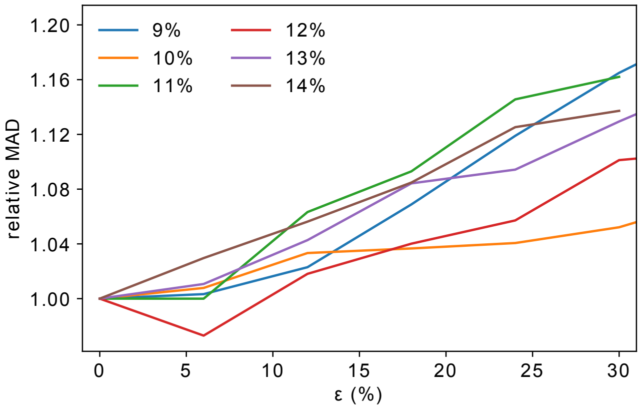

Figure 5. MAD evolution upon straying from single simulations of differerent crystallinities for fibers with small crystals ($1.9 \times 2 \times 2.7\,\mathrm{nm}^3$) and 0° tilt angle against the fiber axis.

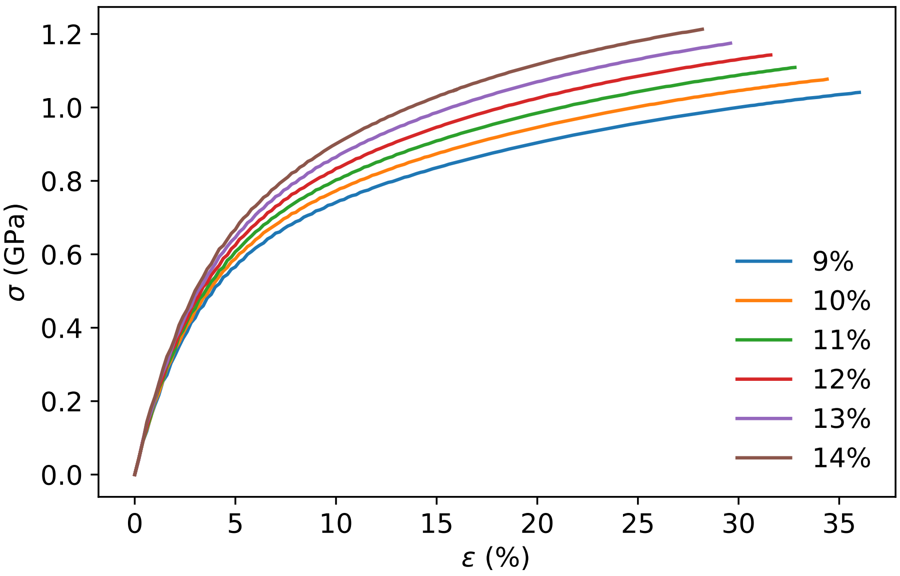

Figure 6. Representative stress-strain curves obtained from FE calculations of fiber models with varying crystallinity. Due to not having finite length chains, our simplified FE model does not capture the strain induced hardening for high strain values as observed in experiments.

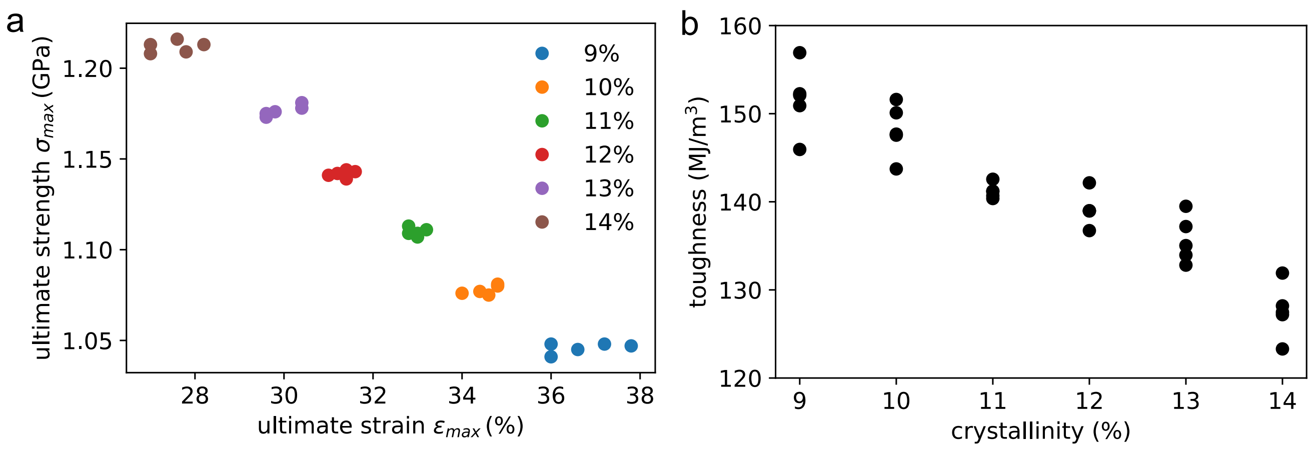

Figure 7. Mechanical properties for fiber models with different crystallinity. (a) Rupture strain and stress, and (b) toughness.

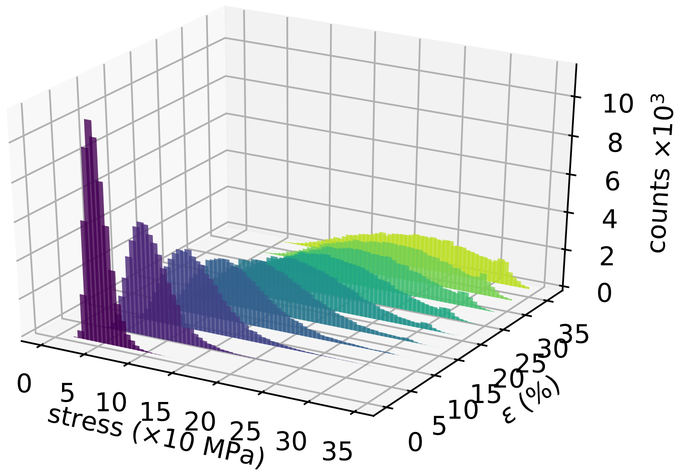

Figure 8. Histograms of von Mises-stresses in all crystal nodes for varying strains. Data for one fiber model with 11 % crystallinity is shown. We defined fiber rupture when more than 5 % of crystals are stressed beyond the defined yield stress of 0.3 GPa, which here results in a rupture strain of 32.8 %.

Supplementary Tables

Table 1. Summary of the mechanical properties from simulations of the 3D FE fiber model and from experimental rheology data.

mechanical properties	3D FE fiber model	experimental data[a]
ultimate strength, σ_{max} (GPa)	1.04 – 1.23	0.65 – 1.61
extensibility, ϵ_{max}	0.27 – 0.38	0.23 – 0.45
toughness (MJ/m^3)	128 – 157	120 – 225

[a] Experimental data for *Araneus diadematus* dragline silk are taken from[28,35–38].

References

1. Sapede, D. *et al.* Nanofibrillar structure and molecular mobility in spider dragline silk. *Macromol.* **38**, 8447–8453 (2005).
2. Giesa, T., Arslan, M., Pugno, N. M. & Buehler, M. J. Nanoconfinement of spider silk fibrils begets superior strength, extensibility, and toughness. *Nano letters* **11**, 5038–5046 (2011).
3. Grubb, D. T. & Jelinski, L. W. Fiber morphology of spider silk: the effects of tensile deformation. *Macromol.* **30**, 2860–2867 (1997).
4. Plaza, G. R. *et al.* Relationship between microstructure and mechanical properties in spider silk fibers: identification of two regimes in the microstructural changes. *Soft Matter* **8**, 6015–12 (2012).
5. Cetinkaya, M., Xiao, S., Markert, B., Stacklies, W. & Gräter, F. Silk fiber mechanics from multiscale force distribution analysis. *Biophys. J.* **100**, 1298–1305 (2011).
6. Cetinkaya, M., Xiao, S. & Gräter, F. Effects of crystalline subunit size on silk fiber mechanics. *Soft Matter* **7**, 8142–8148 (2011).
7. Xiao, S., Stacklies, W., Cetinkaya, M., Markert, B. & Gräter, F. Mechanical response of silk crystalline units from force-distribution analysis. *Biophys. J.* **96**, 3997–4005 (2009).
8. Madurga, R. *et al.* Persistence and variation in microstructural design during the evolution of spider silk. *Nat. Publ. Group* 1–11 (2015).
9. *Silk Polymers* (American Chemical Society, Washington, DC, 2009).
10. Geuzaine, C. & Remacle, J.-F. Gmsh: A 3-d finite element mesh generator with built-in pre- and post-processing facilities. *Int. J. Numer. Meth. Eng.* **79**, 1309–1331 (2009).
11. Schöberl, J. Netgen an advancing front 2d/3d-mesh generator based on abstract rules. *Comput. Vis. Sci.* **1**, 41–52 (1997).
12. Patil, S. P., Markert, B. & Gräter, F. Rate-dependent behavior of the amorphous phase of spider dragline silk. *Biophys. J.* **106**, 2511–2518 (2014).
13. Patil, S. P., Gräter, F. & Markert, B. Refining a bottom-up computational approach for spider silk fibre mechanics. *Proc. 3rd GAMM Semin. Continuum Biomech.* **II–21**, 75–87 (2012).
14. Simmons, A. H., Michal, C. A. & Jelinski, L. W. Molecular orientation and two-component nature of the crystalline fraction of spider dragline silk. *Sci.* **271**, 84 (1996).
15. Yang, Z., Grubb, D. T. & Jelinsk, L. W. Small-angle x-ray scattering of spider dragline silk. *Macromol.* **30**, 8254–8261 (1997).
16. Riekel, C. *et al.* Aspects of x-ray diffraction on single spider fibers. *Int. J. Biol. Macromol.* **24**, 179–186 (1999).
17. Krasnov, I. *et al.* Strain-dependent fractional molecular diffusion in humid spider silk fibres. *J. R. Soc., Interface* **13**, 20160506 (2016).
18. Lewis, R. V. Spider silk: the unraveling of a mystery. *Acc. Chem. Res.* **25**, 392–398 (1992).
19. Hijirida, D. H. *et al.* 13c nmr of nephila clavipes major ampullate silk gland. *Biophys. J.* **71**, 3442 (1996).
20. Dicko, C., Knight, D., Kenney, J. M. & Vollrath, F. Secondary structures and conformational changes in flagelliform, cylindrical, major, and minor ampullate silk proteins. temperature and concentration effects. *Biomacromolecules* **5**, 2105–2115 (2004).
21. Lefevre, T. *et al.* Conformation of spider silk proteins in situ in the intact major ampullate gland and in solution. *Biomacromolecules* **8**, 2342–2344 (2007).
22. An, B. *et al.* Reproducing natural spider silks' copolymer behavior in synthetic silk mimics. *Biomacromolecules* **13**, 3938–3948 (2012).
23. Bezanson, J., Edelman, A., Karpinski, S. & Shah, V. B. Julia: A fresh approach to numerical computing. *CoRR* **abs/1411.1607** (2014). URL http://arxiv.org/abs/1411.1607. 1411.1607.
24. Hunter, J. D. Matplotlib: A 2d graphics environment. *Comput. Sci. Eng.* **9**, 90–95 (2007). DOI 10.1109/MCSE.2007.55.
25. Carmichael, S. & Viney, C. Molecular order in spider major ampullate silk (dragline): Effects of spinning rate and post-spin drawing. *J. Appl. Polym. Sci.* **72**, 895–903 (1999).

26. Drodge, D. R., Mortimer, B., Holland, C. & Siviour, C. R. Ballistic impact to access the high-rate behaviour of individual silk fibres. *J. Mech. Phys. Solids* **60**, 1710–1721 (2012).

27. Guan, J., Porter, D. & Vollrath, F. Silks cope with stress by tuning their mechanical properties under load. *Polym.* **53**, 2717–2726 (2012).

28. Gosline, J., Guerette, P., Ortlepp, C. & Savage, K. The mechanical design of spider silks: from fibroin sequence to mechanical function. *J. Exp. Biol.* **202**, 3295–3303 (1999).

29. Ulrich, S., Glišović, A., Salditt, T. & Zippelius, A. Diffraction from the β -sheet crystallites in spider silk. *Eur. Phys. J. E Soft Matter* **27**, 229–242 (2008).

30. Paquet-Mercier, F., Lefèvre, T., Auger, M. & Pézolet, M. Evidence by infrared spectroscopy of the presence of two types of β-sheets in major ampullate spider silk and silkworm silk. *Soft Matter* **9**, 208–215 (2013).

31. Work, R. W. & Emerson, P. D. An apparatus and technique for the forcible silking of spiders. *J. Arachnol.* **10**, 1–10 (1982).

32. Seydel, T. *et al.* Increased molecular mobility in humid silk fibers under tensile stress. *Phy. Rev. E Stat. Nonlin. Soft Matter Phys.* **83**, 016104 (2011).

33. Xu, M. & Lewis, R. V. Structure of a protein superfiber: spider dragline silk. *Proc. Natl. Acad. Sci. U.S.A.* **87**, 7120–7124 (1990).

34. Gatesy, J., Hayashi, C., Motriuk, D., Woods, J. & Lewis, R. Extreme diversity, conservation, and convergence of spider silk fibroin sequences. *Sci.* **291**, 2603–2605 (2001).

35. Denny, M. The physical properties of spider's silk and their role in the design of orb-webs. *J. Exp. Biol.* **65**, 483–506 (1976).

36. Köhler, T. & Vollrath, F. Thread biomechanics in the two orb-weaving spiders araneus diadematus (araneae, araneidae) and uloborus walckenaerius (araneae, uloboridae). *J. Exp. Zool.* **271**, 1–17 (1995).

37. Vehoff, T., Glišović, A., Schollmeyer, H., Zippelius, A. & Salditt, T. Mechanical properties of spider dragline silk: Humidity, hysteresis, and relaxation. *Biophys. J.* **93**, 4425–4432 (2007).

38. Work, R. W. The force-elongation behavior of web fibers and silks forcibly obtained from orb-web-spinning spiders. *Textile Res. J.* **46**, 485–492 (1976).
